# Comfort for the Poor

**Published:** 1855-10

**Authors:** 


### COMFORT FOR THE POOR.

How grandly benign the first penal sentence on man—"*In the sweat of thy face shalt thou eat bread*, UNTIL THOU RETURN TO THE GROUND"! How blessed the necessity of having to labor for one's daily food. It is steady, moderate work, which gives health, and years, and honor, and success. And yet, perhaps nine out of ten of us, are looking and laboring for the great consummation of being placed in a position which shall require no labor, the fabled *otium cum dignitate;* forgetting, that in the evasion of the penalty, greater judgments come. The sentence is, we must work *until we die.* That is its meaning and intent, and there is within it blessings in disguise; blessings in our very punishments! greater blessings than if we succeed in escaping them! How incomprehensible, how broadly reaching is the love of HIM, with whom we have to do!

It is within the observation of all of us, that those who retire from a long and active business life, soon fall into disease and die; and scarcely a day passes, in which the observant physician does not see, that the difference to many between health and disease, is the distance between the drawing-room and the wash-tub—the distance between the quill and the crow-bar—while the instructive fact with its terrible warning stares us in the face, that "*the average duration of the life of men after* 'RETIRING FROM BUSINESS' *is less than three years.*"

